# Third Party Cord Blood Transplant Boosts Autologous Hematopoiesis in a Case of Persistent Bone Marrow Aplasia after Double Transplant Failure for B-Thalassemia Major

**DOI:** 10.4084/MJHID.2013.029

**Published:** 2013-04-15

**Authors:** Giuseppe Visani, Paola Picardi, Barbara Guiducci, Federica Loscocco, Claudio Giardini, Moira Lucesole, Sara Barulli, Teresa Ricciardi, Alessandro Isidori

**Affiliations:** Hematology and Hematopoietic Stem Cell Transplant Centre, AORMN Marche Nord Hospital, Pesaro, Italy

## Abstract

A 9-year-old female received an allogeneic stem cell transplant (SCT) from an ABO-incompatible HLA-matched sibling for β-thalassemia major, without achieving a complete donor chimerism. Subsequently, the patient received five donor lymphocyte infusions, without increasing donor chimerism, and autologous SCT. Due to the persistent bone marrow aplasia, the patient received a second allogeneic SCT from the same donor without obtaining any engrafment. After the double transplant failure, we performed an unrelated transplant from a full-matched umbilical cord blood (UCBT) without administering any neither conditioning regimen nor GVHD prophylaxis. Forty days after UCBT, trilinear engraftment was documented. Surprisingly, the hematopoietic reconstitution was related to the re-expansion of the autologous (beta-thalassemic) hematopoietic stem cell, as documented by chimerism studies. At present, 30 months after UCBT, there is stable hematopoietic autologous reconstitution. This is the first description of the restoration of autologous hematopoiesis obtained with UCBT in a thalassemia-major patient after a double transplant failure.

## Introduction

Allogeneic stem cell transplantation (allo-SCT) for major thalassemia patients may be complicated by a relevant rejection rate varying, according to the risk classes and conditioning regimens, from 15 to 35%.^1^ Rejection is particularly frequent in thalassemic patients who receive a conditioning regimen with less than 200 mg/kg cyclophosphamide for a risk class III.^2^ The rejection often appears as a progressive loss of take, as shown by the progressive donor cells reduction in chimerism assays.^1^ For patients having a sibling donor who failed the first transplant, a possible second attempt with allo-SCT should be performed.^2^ However, the second transplant could be unsuccessful, leaving a persistent bone marrow aplasia without autologous reconstitution, with high risk of severe complications and death.^1^,^2^ It is unclear why persistent bone marrow aplasia develops in these conditions. Even if a definite absence of hemopoietic stem cell reservoir has been demonstrated in some cases, due to transplant-related toxicity on the stem cell niche, the immune system probably plays a pivotal role stopping the autologous reconstitution.

## Case Report

**Figure 1:**
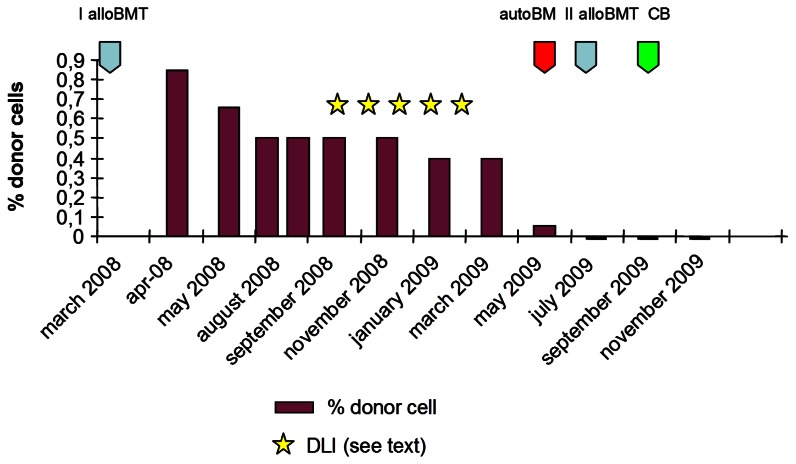
Major clinical events occurred in the patient after an ABO incompatible (donor A Rh+; recipient 0 Rh+) HLA matched sibling for β-thalassemia major.

We report the case of a 9-year-old female patient who received a stem cell transplant from an ABO incompatible (donor A Rh+; recipient 0 Rh+) HLA matched sibling for β-thalassemia major. At the time of transplant (March 2008) the patient was irregularly transfused and was not submitted to a regular chelation therapy. She presented a severe iron overload (ferritin: 1974 ng/mL), defect of factors V and VII and hypoparathyroidism. Liver biopsy revealed a state of moderate parenchymal and mesenchymal hemosiderosis. The conditioning regimen included Hydroxyurea 30 mg/kg (from day −45 to day −12), Busulphan 14 mg/kg (90 mg/day from day −9 to day −6) Cyclophosphamide 160 mg/kg (40 mg/kg/day from day −5 to day −2) and Fludarabine 100 mg/sqm (20 mg/sqm from day −17 to day −13). GVHD prophylaxis consisted of Azathioprine 3 mg/kg (from day – 45 to day −12) and Cyclosporin A (CsA). CsA dosage was initially 3mg/kg/day (from day −2 to day +60), and was then gradually tapered until withdrawn on day +365. A total of 2.68 × 10^8^/kg allogeneic nucleated cells were reinfused. White blood cell recovery (ANC > 0.5 ×10^9^/l) occurred at day +17 after the transplant. Chimerism studies on day + 21 showed a mixed chimerism with prevalence (85%) of donor cells. Subsequent detections showed a progressive reduction of donor chimerism (60% on day + 80; 50% on day +100). (Figure 1) Two months and a half later (+150) reduced mixed chimerism was confirmed (50% of donor cells) and due to this, five increasing dose infusions (from 1×10^4^/kg to 2×10^5^/kg) of donor lymphocytes (DLI) were performed from September 2008 to February 2009, in order to further increase donor chimerism. On the contrary, we observed a further progressive reduction of donor chimerism in both bone marrow and peripheral blood cells, with a complete loss on day + 398 from transplant (40% on day + 270; 5% on day 360). On day + 416 the patient received an autologous reinfusion of marrow stem cells, previously stored as back up (Total Nucleated Cells 2.3 × 10^8^/kg). Afterwards, no increase in peripheral blood cell counts was observed and bone marrow was persistently aplastic. A second bone marrow transplantation, from the same familiar donor, was thus performed 16 months after the first one. The conditioning regimen included Busulphan 6.4 mg/kg (98 mg/die from day – 7 to day −6) and Fludarabine 150mg/sqm (30 mg/sqm/day from day −7 to day −3). A total of 2.05 × 10^8^/kg nucleated cells were reinfused. A bone marrow evaluation was performed on day + 21, revealing a persistent, severe aplasia. Chimerism studies showed a complete recipient chimerism, with the absence of donor-derived hematopoietic cells.

**Figure 2:**
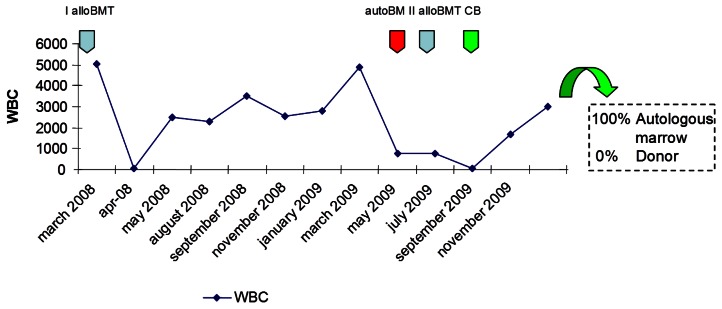
White blood cells count of the patient after transplants.

The double transplant failure prompted us to program an unrelated cord blood transplant (UCBT), that was performed 78 days after the second allo-SCT. The patient was transplanted with a 6/6 matched umbelical cord blood (UCB) (0 Rh+, male). A total of 3×10^7^ TNC/kg were infused. Due to the severe immunosoppression of the patient, as confirmed by the evaluation of circulating lymphocytes (0.01 lymphocytes ×10^9^/l), she did not receive any conditioning regimen nor GVHD prophylaxis. The aim was to obtain a permanent take of cord blood derived hemopoietic cells with recovery of peripheral blood cell counts. On day + 40 after UCBT, WBC count reached 1.68×10^9^/l, platelets were 18×10^9^/l, and hemoglobin 8.8 g/dl. Laboratory tests, surprisingly, showed that the reconstitution was related to the re-expansion of the autologous (beta thalassemic) hemopoietic stem cells. In fact, chimerism studies on both peripheral blood and bone marrow showed a complete recipient chimerism, with the absence of donor-derived hematopoietic cells (Figure 1 and Figure 2).

More than 30 months after the unrelated cord blood transplant, there is stable hemopoietic autologous reconstitution. Chimerism studies performed every 6 months showed a full recipient chimerism, with the absence of donor-derived hematopoietic cells of sibling or cord blood origin. The patient is followed in the outpatient setting, supported with packed red blood cells, similarly to the pre-transplant period. To the best of our knowledge, this is the first description of the restoration of autologous hematopoiesis after a double transplant failure obtained in a patient affected with thalassemia major following cord blood infusion.

Cord blood has been shown to harbor a wide variety of precursor cells, ranging from oriented hemopoietic precursor cells, to mesenchymal stem cells (MSC), to more immature elements, some still maintaining primitive self-renewal capacity.^3^ It is well known that third party mesenchymal stem cells, that were recently isolated also from cord blood^4^, could play a “bystander effect” in transplant situations^5^, favoring the take of donor cells, or modulating the interactions between host and graft^5^. A bystander effect is, in fact, the secondary effects on adjacent cells and tissues triggered by treatment of a primary target with a therapeutic agent.

We think that, in this anedoctical observation, it is possible that third party cord blood cells exerted a “bystander effect”, boosting the activation of autologous hematopoiesis and, therefore, restoring a sustained production of mature cells, thus avowing the probable aplasia related death. This observation was further supported by the fact that we were not able to demonstrate, with mixed chimerism assays, the reconstitution of cord blood-derived hematopoiesis in this patient, prompting us to consider the possible “bystander effect” role of the cord blood cell^4^. As a matter of fact, a “bystander effect” of a cord blood unit has been postulated also for the co-transplant of two different cord blood units,^6^ where one of the two units favors the take, disappearing afterwards, with no apparent contribution to the effective hematopoiesis after transplant.^6^

A further example of successful recovery of bone marrow function after transplant has been reported by Fouillard et al., infusing allogeneic-related HLA mismatched mesenchymal stem cells, as treatment of bone marrow failure following autologous hemopoietic stem cell transplantation, in a patient affected by acute leukemia.^7^ Hematopoietic recovery was observed with a parallel improvement of in vitro clonogenic assay and detection of allogeneic MSC in recipient bone marrow. Notably, even if the place of MSC in the treatment of bone marrow failure is unknown, some in vitro and animal data are in favor of a possible therapeutic role. Improvement of the bone marrow microenvironment by MSC was demonstrated in a patient with end stage severe aplastic anemia.^8^

Cord blood, anyway, is known for presenting a quite small number of MSC,^9^ even if harboring a wide number of different precursor cells (including MSC). It is thus difficult to postulate the successful reactivation of autologous hemopoietic production occurred in this patient with the sole effect of cord blood MSC. Thus, the possible “bystander effect” performed by UCBT in this patient could be, almost in part, related to immunological interactions, able to reverse the microenvironment inhibition previously responsible for the suppression of autologous hematopoiesis.^10^

In conclusion, thalassemia major patients with persisting severe aplastic anemia due to multiple allo-SCT failure are in life-threatening situation with only few therapeutic options available. Even if there is no proven evidence, our anedoctical observation suggest that third party UCBT could probably exert a “bystander effect” able to boost re-expansion of the autologous (beta-thalassemic) hematopoietic stem cell, leading to the restoration a sustained hematopoietic recovery, with the consequent resolution of the life-threatening situation.
